# Desistance from crime following substance use treatment: the role of treatment retention, social network and self-control

**DOI:** 10.1186/s12888-021-03518-2

**Published:** 2021-11-12

**Authors:** Ingeborg Skjærvø, Thomas Clausen, Svetlana Skurtveit, Anne Bukten

**Affiliations:** 1 Norwegian Centre for Addiction Research (SERAF), Faculty of Medicine, University of Oslo, Oslo, Norway; 2 Department of Research and Development in Mental Health Service, Akershus University Hospital, Lørenskog, Norway; 3 Department of Mental Disorders, Norwegian Institute of Public Health, Oslo, Norway

**Keywords:** Substance use treatment, Treatment retention, Desistance, Crime, Stimulants, Amphetamine, Opioid maintenance treatment, Social network, Self-control

## Abstract

**Background:**

Reductions in crime are often reported following substance use treatment. We explore the relationship between desistance from crime, treatment type, treatment retention and positive changes in known risk factors for crime.

**Methods:**

We used data from the NorComt-study; a longitudinal study of substance users (*n* = 341) enrolled in comprehensive treatment in Norway (2012–2015). At treatment initiation (T0) and 1 year later (T1), we collected self-reported data on criminal involvement, treatment, substance use, social network and self-control. We calculated adjusted odds ratios (aOR) and 95% confidence intervals (CI) with multinomial logistic regression analysis.

**Results:**

Overall, 1 year following treatment initiation 69% reported desistance from crime, 18% reported continued crime and 12% reported no crime at all in the study period. Desistance was high for OMT patients in ongoing treatment (79% desisted) and for inpatients regardless of treatment status (79–93% desisted), while not as high among OMT patients with interrupted treatment (47% desisted). For participants that continued crime during follow-up, the average number of criminal acts per month was reduced (*p* < 0.001). Desistance at follow-up was associated with being older (aOR: 1.05, CI: 1.00–1.10), inpatient treatment (aOR: 3.71, CI: 1.12–12.29), being in ongoing treatment (inpatient or OMT) (aOR: 2.90, CI: 1.01–8.36), having no stimulant use in the study period (aOR: 4.86, CI: 1.72–13.70), leaving a substance using social network (aOR 2.87, CI: 1.15–7.18) and improvement in self-control score (aOR: 1.08, CI: 1.04–1.13).

**Conclusions:**

Retention in treatment is particularly important for crime outcomes among OMT patients. Positive changes in social network and self-control are potential contributors to desistance from crime. Targeted interventions towards crime reduction are recommended for patients with stimulant use, which appears to be a persistent risk factor for crime over time.

**Supplementary Information:**

The online version contains supplementary material available at 10.1186/s12888-021-03518-2.

## Background

The link between substance use and crime is well established. Before substance use treatment, 40 to 60% of substance users self-report recent criminal activity [1–5]. Clear reductions in self-reported criminal offending have been found 1 year after initiation of opioid maintenance treatment (OMT) [2, 6–8] or inpatient treatment [2, 7, 9].

There are several overlaps between the factors and prerequisites that are considered necessary for recovery from substance use, and those that are considered necessary for desistance from crime [10]. Criminally involved substance users often consider dependence their main problem and see desistance from crime as a natural result of recovery from their dependence [11]. Studies show that substance use treatment and treatment engagement are central factors for reducing crime among substance users [2, 12–14] and that reductions in substance use may mediate the effect of substance use treatment on crime [15, 16]. At the same time, focus on criminal involvement in substance use treatment could be important, as criminal involvement and criminal networks can be barriers for substance use recovery [3].

Recovery from substance use is often a means to an end. One of the underlying goals that can drive change in substance use is related to improving social and family networks [11, 17]. At the same time, social and family networks can be motivators for and facilitators of change, both when it comes to recovery from substance use [18, 19] and desistance from crime [20–22]. Changes in social identity and in group membership is considered central for the processes of recovery and desistance [10]. These changes could involve for example moving from an identity as a “successful criminal” to “family man” and changing belonging from a social network that accepts antisocial and unlawful actions to a network that provides more positive values and social resources. In this context, it is interesting that inclusion of social skills training in treatment, which could help patients achieve better social relationships, has had promising effects on treatment outcomes [23].

Among individual-level factors, self-control is relevant for several reasons. First, self-control has been considered a central part of understanding crime since the self-control theory of crime was proposed [24, 25], although more recent elaborations of the theory may be more useful in defining the future role of self-control research in crime prevention [26]. Second, there is an established relationship between substance use and self-control, often in the form of disinhibition or impulsivity. Self-control has been implicated in explaining and predicting substance use [27–31], and there is evidence that substance use disrupts the neurological circuits that underlie self-control [31, 32]. Recently, the possibility of change in trait self-control has been highlighted [26, 33]. In sum, there is varied evidence that implicates change in self-control as an important target when seeking recovery from substance use dependence and desistance from crime.

Increased knowledge of social and individual level changes for patients with positive crime outcomes may be helpful in tailoring treatment for substance users also involved in crime and thus contribute to better substance use and crime outcomes. In a previous paper, we found that criminal activity before treatment initiation was associated with higher levels of polysubstance use and stimulant use, having a substance using primary social network and lower self-control [5]. In this study, with longitudinal data from the same population, we investigate how changes in criminal activity 1 year after treatment initiation were related to treatment status and these previously identified factors.

### Aims

The aims were to 1) Investigate desistance from crime 1 year after initiation of substance use treatment, by gender, treatment type and treatment status, and 2) explore how treatment status and change in substance use, social network and self-control were related to desistance 1 year after treatment initiation.

## Methods

### Design

NorComt (Norwegian Cohort of Patients in Opioid Maintenance Treatment and Other Drug Treatment) is a longitudinal multi-site study, with patients from 14 OMT-centres and 7 inpatient facilities across Norway. Baseline data (T0) were collected at treatment initiation (December 2012–March 2015), and “one-year” follow-up data (T1) were collected after 11–18 months.

### Setting

Substance use treatment is available at no cost through Norway’s publicly funded healthcare, and applications for treatment are mediated through social services or medical practitioners. The general evaluation process and criteria for allocation of treatment in Norway have been described previously [5, 34]; in short, a regional team selects patients’ towards appropriate treatment based on the severity of substance use, general situation and the expected benefit and cost of treatment. For patients that need comprehensive substance use treatment, OMT or inpatient treatment would usually be the appropriate options. The treatment centres included in this study treat patients with use of illicit substances although use of alcohol and prescribed addictive substances co-occur for many. Patients enrolled in treatment in Norway are mainly polysubstance users [35].

### Procedure and participants

Treatment centre staff conducted the baseline interviews and reported a total of 1415 patients starting treatment in the study-period. However, 670 of these patients were never approached for participation. The treatment centres logged the following reasons for not approaching participants: insufficient staff resources (47%), reason not reported (33%), early discharge (9%), mental health status (7%), severity of substance use (3%), physical health status (< 1%) and language barriers (< 1%). Three of the 21 treatment centres did not have resources to prioritize study participation. These were OMT centres in three major cities in Norway, who through the overall size of their catchment areas and patient flow contributed over half of the patients that were never approached for participation, while simultaneously contributing the majority of the OMT patients in the study.

Of the 745 that were approached for participation, 548 (74%) signed the informed consent and completed the baseline interview (T0) within 12 weeks of treatment initiation (median 18 days), 17% declined and 6% missed the appointment/s for participation.

Agreeing to a follow-up interview was not a criterion for inclusion at baseline, however participants consented to be contacted again and provided contact information for themselves and for example family, friends or social services.

Of the 548 interviewed at T0, 62% (*n* = 341) were re-interviewed at T1. The goal was to naturalistically follow a cohort of patients that entered substance use treatment, with no exclusion criteria related to treatment retention or completion. The follow-up rate (62%) can appear low, however it has been found that data from 60% of substance use patients are as representative of the full population as 90–100% of the patients [36]. Altogether 38% of the patients did not complete the follow-up questionnaire; 12% could not be reached after repeated efforts, 11% declined, 9% did not make it to appointment/s, 5% were lost due to logistical challenges/errors with the research group and 1% were confirmed deceased. The median time between T0 and T1 was 14.5 months.

We used unpaired t-tests and chi-square tests to compare characteristics of participants that were re-interviewed at follow-up with those who were lost to follow-up. Although there was no difference in criminal involvement (yes/no) in the 6 months before treatment initiation, the re-interviewed had committed a higher number of crimes. There were no differences in other relevant variables at baseline (see Supplementary Table 1, Additional File 1).

Of the included participants, 179 (mean age 39 years, 27% women) started OMT and 162 (mean age 29 years, 31% women) started inpatient treatment. At follow-up, the initial treatment was ongoing for 89% of OMT patients and interrupted for 11%. Among inpatients, the treatment was ongoing for 18%, interrupted for 35% and completed for 47%. Both treatment groups were mainly polysubstance users at baseline (88% of inpatients with a mean of 5 different substances and 84% of OMT patients with a mean of 4 different substances). Stimulant use was of particular interest in this study; 82% of inpatients and 54% of OMT patients reported stimulant use at baseline (Supplementary Fig. 1, Additional File 1).

### Measures

#### Outcomes

The main outcome variable was self-reported *change in crime status (desisted/no crime/continued crime)* from T0 to T1. Participants were asked “How would you describe your criminal activity *now* compared to *before* treatment?” with five response-options. We collapsed these responses into 3 categories: Desisted (“no crime anymore”), no crime (“not applicable”) and continued crime (“reduced”, “no change” and “increased”).

*Change in type and number of crimes* was measured by asking about crime during two time-periods: during the 6 months before T0 and during the 12 months before T1. To address this difference in time-period, we calculated a monthly crime rate for each participant by dividing the number of acts by the corresponding number of months (6 months at baseline and 12 months at follow-up). We asked participants about 5 subcategories of crime (acquisitive, substance related, violent, traffic violations or other – possession and use of illegal substances were excluded as a criminal offence in this context). Of those that reported crime during both time-periods, 63 (70%) provided valid data on the number of criminal acts in the respective time-periods.

#### Independent variables

Treatment status at T1: Participants were categorized as treatment completed according to plan, interrupted treatment (voluntary or involuntary), or in ongoing treatment at the time of follow-up (T1).

Change in number of substances used: Participants were asked about the number of different substances they had used in the 6 months before T0 and the 6 months before T1 [37]. The number of substances reported at T0 was then deducted from the response at T1 for each individual, giving a continuous variable reflecting reduction in poly-substance use.

Change in stimulant use: Participants were asked to list their four most used substances or addictive medications in the past 6 months before T0 and T1 [37]. Participants were considered users of stimulants if it was among their four most used in the past 6 months. Amphetamines, cocaine and other stimulants were combined into the category “stimulants”. Participants were categorised as having continued use (T0: yes/no, T1: yes), ended use (T0: yes, T1: no) or not used in the study period (T0: no, T1: no).

Change in primary social network: We used a question from EuropASI [38] concerning the last 6 months: “With whom do you usually spend most of your free time?” Response options were family or friends, with or without problem use of alcohol, medications and substances, or being mostly alone. Responses were dichotomized into having a primarily substance using social network (family or friends with problem substance use), or not (family or friends without problem substance use, or being mostly alone) [39]. Participants were placed in 3 categories to reflect change in their social network from T0 to T1: Continued substance using network (T0: yes/no, T1: yes), left substance using network (T0: yes, T1: no) and no substance using network in the study period (T0: no, T1: no).

Change in self-control score: The Brief Self Control Scale (BSCS) reflects impulse control and self-discipline/restraint [40, 41]. It is a validated scale consisting of 13 items with a 5-point Likert response-scale (1 to 5). The summed score ranges from 13 to 65 (low to high self-control) [42]. Participants with > 2 missing items were excluded from analysis (*n* = 7) and we imputed the individual person mean for participants with only 1 or 2 missing items (*n* = 36) [43, 44]. Internal consistency of the scale was high with Cronbach’s α = 0.83 at T0 and α = 0.84 at T1. We subtracted the score at T0 from the score at T1 to create a continuous variable reflecting change in self-control. A negative value reflects the magnitude of reduction in the score and a positive value reflects the magnitude of improvement.

### Analysis strategy

Of the 341 participants that completed T1, 334 had valid data on the main outcome variable and were included in analyses. We investigated the difference between the three crime groups using univariate ANOVA and Chi-Square tests. Significant omnibus-test were followed up with pairwise tests, respectively Tukey’s Honestly Significant Difference test (Tukey’s HSD) or Bonferroni-corrected z-tests (corrected within each set of analyses). In the multinomial logistic regression, the outcome variable was change in crime status at T1, with the continued crime group as reference group. The adjusted model included age, gender, type of index treatment at T0 (inpatient or OMT), index treatment status at T1, change in number of substances used, change in stimulant use, change in social network and change in level of self-control. All preliminary analyses were done for the inpatient group and OMT group separately. As all results were in the same direction, we kept the sample as one group of patients enrolled in comprehensive substance use treatment, while controlling for type of treatment in the analyses. We used the Wilcoxon signed rank test to compare the average number of monthly criminal acts 6 months before T0 and 12 months before T1. The level for statistical significance was 5% for all tests. We used IBM SPSS 25 for statistical analyses.

## Results

### Changes in crime by treatment type and treatment status

**Fig. 1:**
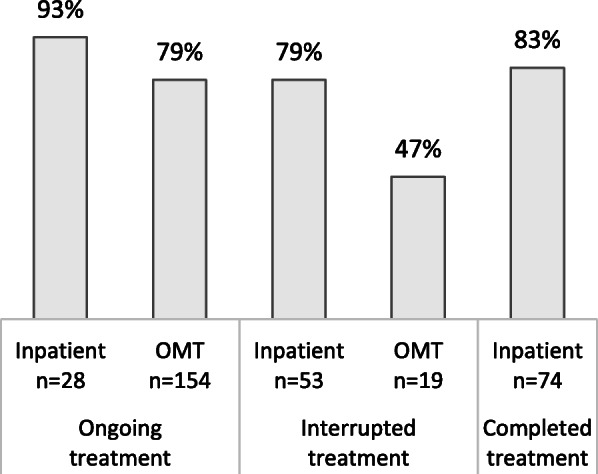
Desistance at T1 divided by treatment type and treatment status (*n* = 334)

At the time of follow-up, 69% (*n* = 232) reported desistance from crime, 18% (*n* = 61) reported continued crime (within the continued crime group: 14% reduced, 3% unchanged, 1% increased crime), while 12% (*n* = 41) reported no crime in the study period. There were high rates of desistance across treatment type and status, except for OMT patients with interrupted treatment (Fig. 1).

When asked about type and number of crimes during the 6 months before T0 and the 12 months before T1, reductions were seen across all categories of crime (acquisitive, substance-related, violence, traffic-violations and other: see Supplementary Fig. 2, Additional File 1). In total, participants had committed 907 acts per month (mean: 14.4, median: 4.0, per person) in the 6 months before T0, and 281 acts per month (mean: 4.5, median: 0.4, per person) in the 12 months before T1. This reduction was statistically significant (Wilcoxon signed rank test: Z = -4.07, *p* < 0.001). When stratifying by treatment type and treatment status, a reduction was observed in all subgroups, although most pronounced among inpatients (see Supplementary Fig. 3, Additional File 1). Patients with interrupted treatment and inpatients in ongoing treatment committed a higher number of crimes at baseline compared to inpatients that had completed treatment and OMT patients that were in ongoing treatment. This stratification is exploratory and the n in each subgroup is too small to conduct meaningful statistical tests for significant differences.

### Comparisons of desisted, continued and no crime groups

When comparing the crime groups (Table 1), the no crime group was on average more than 10 years older than the two other groups (*p* < 0.001 for both comparisons) and contained a higher proportion of women compared to the continued crime group (*p* = 0.025). Only 10% of the no crime group were inpatients, which was less than in the desisted crime (*p* < 0.001) and continued crime groups (*p* = 0.001). At the same time, OMT patients were more likely to be in the no crime group compared to the desisted crime (*p* < 0.001) and continued crime groups (p = 0.001). Looking at treatment status for inpatients, there was no statistically significant difference for the crime groups: interrupted treatment was equally common for those who continued and those who desisted crime. Among OMT patients, a larger proportion of the continued crime group had interrupted treatment (26%) compared to the desisted crime (8%, *p* = 0.013) and no crime (5%, *p* = 0.043) groups.

**Table 1:** Treatment factors and changes in substance use, social network and self-control for three crime groups

	No crime T0	Crime T0			*p*-value ^b^
	No crime T1^a^ *n* = 41	Continued T1 *n* = 61	Desisted T1 *n* = 232	F or X^2^ value (df)	
**Demographics**					
Age at baseline, mean (median)	44.5 (45.1)	32.0 (29.8)	32.6 (30.6)	F = 31.17 (2)	***p*** **< 0.001**
Women, n (%)	18 (44)	12 (20)	67 (29)	X^2^ = 7.00 (2)	***p*** **= 0.030**
**Treatment**					
Index treatment: Long-term inpatient (ref: OMT)	4 (10)	27 (44)	128 (55)	X^2^ = 29.15 (2)	***p*** **< 0.001**
Treatment status, n (%)					
* Full sample* (*n* = 334)					
Interrupted treatment	2 (5)	20 (34)	50 (22)		
Ongoing treatment at T1	36 (88)	27 (46)	119 (52)	X^2^ = 23.91 (4)	***p*** **< 0.001**
Completed according to plan	3 (7)	12 (20)	59 (26)		
* Inpatients* (*n* = 159)					
Interrupted treatment	3 (75)	12 (48)	59 (47)		
Ongoing treatment at T1	1 (25)	2 (8)	25 (20)	Fisher’s Exact^e^	*p* = 0.30^e^
Completed according to plan	0 (0)	11 (44)	42 (33)		
* OMT-patients* (*n* = 175) ^c^					
Interrupted treatment	2 (5)	9 (26)	8 (8)		
Ongoing treatment at T1	35 (95)	25 (74)	94 (92)	Fisher’s Exact^e^	***p*** **= 0.010**^e^
**Substance use**					
Reduction in number of substances, mean (median)	−1.03 (−1.0)	−0.81 (−1.0)	−2.86 (−2.0)	F = 12.16 (2)	***p*** **< 0.001**
Stimulant use^d^, n (%)					
Continued use	7 (17)	37 (61)	62 (27)		
Ended use	9 (22)	17 (28)	110 (47)	X^2^ = 51.85 (4)	***p*** **< 0.001**
Not used in study period	25 (61)	7 (12)	60 (26)		
**Other factors**					
Change in primary social network, n (%)					
Continued substance using network	3 (7)	26 (43)	40 (17)		
Left substance using network	10 (24)	17 (28)	101 (44)	X^2^ = 34.21 (4)	***p*** **< 0.001**
No substance using network in study period	28 (68)	18 (30)	90 (39)		
Improvement self-control score (BSCS), mean (median)	3.6 (4.0)	1.0 (0.0)	8.9 (9.0)	F = 17.27 (2)	***p*** **< 0.001**

*N* = 334. Significant *p*-values are marked in bold. **Missing data**: Treatment status, *n* = 6; social network, *n* = 1; self-control score, *n* = 6; change in number of substances, *n* = 9. ^a^ No crime in the study period (T0 nor T1). ^b^ One-way ANOVA or Chi-Square tests compare the no crime, continued and desisted crime groups. ^c^ OMT treatment is long-term, thus no patients had completed and left treatment according to plan within the follow-up period. ^d^ Stimulants consists of amphetamines (T0: 88% T1: 93%), cocaine (T0: 22% T1: 11%), crack (T0: 0% T1: 0%), and other stimulants (T0: 5% T1: 3%). ^e^ Fisher’s Exact test

The desisted crime group had a larger reduction in number of substances used compared to the no crime (*p* = 0.004) and continued crime groups (*p* < 0.001). Continued stimulant use was most common in the continued crime group, compared to the no crime (*p* < 0.001) and desisted crime groups (*p* < 0.001). Ending stimulant use was more common in the desisted crime group compared to the no crime group (*p* = 0.007) and the continued crime group (*p* = 0.018). No use of stimulants in the study period was more common in the no crime group compared to the desisted crime (*p* < 0.001) and continued crime group (*p* <  0.001).

**Fig. 2:**
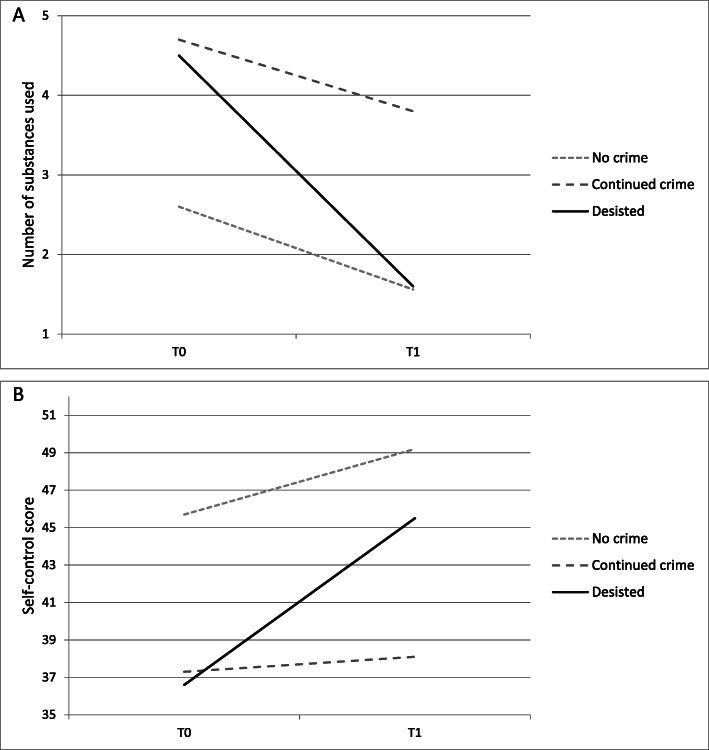
Change in **A:** number of substances used. **B:** self-control score, by crime status at follow-up. *n* = 334. Self-control was measured with the brief self-control scale (BSCS), which ranges from 13 (low) to 65 (high). Number of substances used: We asked about the last 6 months before treatment start (T0) and follow-up (T1).

Having had a mainly non-substance using network throughout the study period was most common in the no crime group compared to the desisted crime (*p* = 0.001) and continued crime group (*p* < 0.001). The continued crime group was more likely to have a substance using social network at follow-up (43%) compared to the desisted (17%, *p* <  0.001) and no crime groups (7%, *p* <  0.001). The desisted crime group had the largest improvement in self-control score, improving their score by more than twice that of the no crime group (*p* = 0.006) and nine times that of the continued crime group (*p* <  0.001). A visualisation of the differences in change from T0 to T1 is presented in Fig. 2: The continued crime group showed little change in number of substances used (Fig. 2A) and self-control (Fig. 2B), while the desisted crime group showed positive changes which made them more comparable to the no crime group. A similar pattern was seen for continued stimulant use and continued substance using social network (Table 1).

### Factors associated with desistance and no crime, compared to continued crime

In the adjusted regression model comparing the *desisted* and continued crime groups (Table 2), desistance was significantly associated with being older (aOR: 1.05, CI: 1.00–1.10), inpatient treatment (aOR: 3.71, CI: 1.12–12.29), being in ongoing treatment (aOR: 2.90, CI: 1.01–8.36), having no stimulant use in the study period (aOR: 4.86, CI: 1.72–13.70), leaving a substance using social network (aOR: 2.87, CI: 1.15–7.18) and improvement in self-control score (aOR: 1.08, CI: 1.04–1.13).

**Table 2:** Multinomial regression analysis where the dependent variable was change in crime status at follow-up

	Desisted vs. Continued crime				No crime vs. Continued crime ^b^			
	Unadjusted OR (95% CI), *n* = 293	*p*-value	Adjusted model OR (95% CI), *n* = 277^a^	*p*-value	Unadjusted OR (95% CI), *n* = 102	*p*-value	Adjusted model OR (95% CI), *n* = 92^a^	*p*-value
**Demographics**								
Age at baseline	1.01 (0.98–1.04)	*p* = 0.64	**1.05 (1.00–1.10)**	***p*** **= 0.041**	**1.14 (1.09–1.19)**	***p*** **< 0.001**	**1.14 (1.07–1.21)**	***p*** **< 0.001**
Being female	1.66 (0.83–3.31)	*p* = 0.15	1.05 (0.45–2.45)	*p* = 0.92	**3.20 (1.32–7.72)**	***p*** **= 0.010**	2.44 (0.77–7.69)	*p* = 0.13
**Treatment**								
Index treatment: Long-term inpatient (ref: OMT)	1.55 (0.88–2.73)	*p* = 0.13	**3.71 (1.12–12.29)**	***p*** **= 0.032**	**0.14 (0.04–0.43)**	***p*** **= 0.001**	0.86 (0.07–10.66)	*p* = 0.91
Treatment status (ref: interrupted treatment)								
*- Ongoing treatment at T1*	1.76 (0.91–3.43)	*p* = 0.10	**2.90 (1.01–8.36)**	***p*** **= 0.049**	**13.33 (2.87–62.00)**	***p*** **< 0.001**	6.30 (0.93–42.88)	*p* = 0.06
*- Completed according to plan*	1.97 (0.88–4.42)	*p* = 0.10	0.53 (0.17–1.64)	*p* = 0.27	2.50 (0.36–17.17)	*p* = 0.35	1.71 (0.11–25.66)	*p* = 0.70
**Substance use**								
Reduction in number of substances used	**1.23 (1.11–1.36)**	***p*** **< 0.001**	1.09 (0.97–1.22)	p = 0.15	1.02 (0.90–1.17)	*p* = 0.73	0.90 (0.73–1.11)	*p* = 0.32
Stimulant use (ref: continued use)								
*- Ended use*	**3.86 (2.01–7.42)**	***p*** **< 0.001**	1.78 (0.71–4.49)	*p* = 0.22	2.80 (0.89–8.77)	*p* = 0.08	**5.38 (1.11–26.11)**	***p*** **= 0.036**
*- Not used in study period*	**5.12 (2.12–12.36)**	***p*** **< 0.001**	**4.86 (1.72–13.70)**	***p*** **= 0.003**	**18.9 (5.89–60.46)**	***p*** **< 0.001**	**11.98 (2.80–51.21)**	***p*** **< 0.001**
**Other factors**								
Change in primary social network (ref: Continued substance using network)								
*- Left substance using network*	**3.86 (1.89–7.88)**	***p*** **< 0.001**	**2.87 (1.15–7.18)**	***p*** **= 0.024**	**5.10 (1.22–21.25)**	***p*** **= 0.025**	**12.60 (2.34–67.88)**	***p*** **= 0.003**
*- No substance using network in study period*	**3.25 (1.60–6.59)**	***p*** **= 0.001**	2.18 (0.93–5.11)	*p* = 0.07	**13.48 (3.55–51.16)**	***p*** **< 0.001**	**9.71 (2.10–44.99)**	***p*** **= 0.004**
Improvement in self-control score (BSCS)	**1.09 (1.05–1.12)**	***p*** **< 0.001**	**1.08 (1.04–1.13)**	***p*** **< 0.001**	1.03 (0.99–1.07)	*p* = 0.20	1.04 (0.98–1.11)	*p* = 0.22

*N* = 334. *OR* Odds ratio. *aOR* Adjusted odds ratio. *CI* Confidence interval. Significant ORs (*p* < 0.05) are in bold. **Missing data**: Treatment status, *n* = 6; social network, *n* = 1; self-control score, *n* = 6; number of substances, *n* = 9. ^a^ 92% (*n* = 315) of participants had valid responses for all variables in the adjusted model. ^b^ Findings from the additional comparison of No crime vs Continued crime should be considered preliminary as the smaller sample size (n = 92) results in wide confidence intervals (CIs) and uncertain estimates

**Model fit:** According to the likelihood ratio chi-square test, the multinomial regression model is a significant improvement in fit compared to the null model (X^2^(22)=137.0, *p* < 0.001). The Pearson’s chi-square goodness-of-fit test also indicated a good model fit (X^2^(606) = 584.6, *p* = 0.73)

In the adjusted regression model comparing the *no crime* to the continued crime group (Table 2), no crime was significantly associated with older age (aOR: 1.14, CI: 1.07–1.21), ended stimulant use (aOR: 5.38, CI: 1.11–26.11), no stimulant use in the study period (aOR: 11.98, CI: 2.80–51.21), having left a substance using social network (aOR: 12.60, CI: 2.34–67.88) and having no substance using network in the study period (aOR: 9.71, CI: 2.10–44.99).

## Discussion

A large proportion of patients had desisted from crime 1 year following treatment initiation. Among those who continued their criminal involvement, there was still a significant reduction in the number of crimes committed. Our results indicate that whether treatment was interrupted, completed or ongoing was an important factor for desistance among OMT patients, but not necessarily among inpatients. Further, desistance was more likely for participants who during the study period had not used stimulants, left a substance using social network and improved their self-control score**.** In sum, this study confirms a link between desistance and recovery-related factors, such as treatment completion and retention, reduction in substance use, and changes in social network and self-control. On the other hand, the role of treatment completion and retention may vary depending on treatment type.

### Treatment and desistance

Although reductions in crime following substance use treatment in itself is not a novel finding [2, 6–9, 45, 46], we found several relationships between treatment factors and crime.

First, even among participants that continued criminal involvement, there was a significant reduction in the number of crimes committed. This underlines the importance of looking beyond complete desistance when investigating the effect of interventions on crime.

Second, inpatients had a higher prevalence of criminal involvement before treatment start. This could be related to the younger age in this patient group or the reported differences in substance use pattern, e.g. more polysubstance use and stimulant use. In adjusted analyses, inpatients were also more likely to desist compared to OMT patients. This could be a result of the described group differences between inpatients and OMT patients (such as substance use pattern), or it could be related to differences in treatment content.

Third, ongoing treatment was associated with desistance, which is in line with several previous studies [12, 47]. However, the importance of treatment status may vary with treatment type. For inpatients, desistance rates were high regardless of treatment status, while nearly half of OMT patients with interrupted treatment continued crime. This could be due to differences in the patients that seek the different treatment types, but also due to differences in the two treatments. Inpatient treatment involves patients being physically removed from their previous day-to-day lives, patterns and social contexts [48]. This treatment-intensity could contribute to a ‘flying start’ for changes in behavioural patterns, including criminal involvement. It is also possible that seeking inpatient treatment reflects a high motivational state, and that some treatment goals were achieved even when treatment was interrupted [49]. OMT patients, on the other hand, typically receive outpatient treatment, often life-long, without immediate major changes in their daily lives or surroundings. When OMT was interrupted early, many patients may have found themselves in a very similar overall situation as when they entered treatment, resulting in a return to old patterns when it came to both substance use and crime. An unanswered question is whether this positive effect of inpatient treatment despite interruption will remain over time, given the relatively short follow-up period of this study.

Fourth, criminal involvement before treatment initiation may also affect treatment retention. OMT patients with no crime in the study period were more likely to be in ongoing treatment after 1 year, and participants with interrupted treatment had committed a higher number of crimes before treatment. It is possible that criminal involvement is a marker for a more severe over all situation for the participants, however, it has also been suggested that aspects of a criminal lifestyle can affect treatment engagement negatively [50]. In the latter case, crime specific interventions may improve outcomes of substance use treatment for some patients.

### Substance use pattern, social network and self-control

Overall, we see that participants who continued crime showed little change in substance use, social network and self-control. Participants that desisted, on the other hand, had positive changes and more closely resembled the no crime group at follow-up. This reflects both the possibility of positive change in these crime-related factors and how these changes co-occur with desistance from crime.

When controlling for a number of other substances, stimulant use has previously been associated with crime among substance users both in and out of treatment [5, 51]. We found that participants with no use of stimulants in the study period were more likely to have desisted from crime or to be in the no crime group. There may be several reasons for the relationship between stimulant use and crime. Amphetamines, the most commonly used stimulant in this sample, can cause irritability, agitation, paranoid states, disorientation and compulsive behaviours [52]. Subgroups of stimulant users have been found to be more risk-taking and sensation-seeking [53], to be more impulsive/disinhibited [53, 54], and to show impaired decision making [55]. One study found poor quality decision making among chronic stimulant users, but not chronic opiate users [56]. A pilot-study found stimulant users to have reductions in loss aversion, which could lead to disadvantageous decision making [57]. It is possible that these traits or behaviours can be part of the explanation of the link between stimulant use and crime (risking the negative consequences of crime), whether the traits were present prior to stimulant use or emerged as a pharmacological effect (acute or degenerative) of the stimulant use. Stimulant users in treatment may thus need targeted interventions to improve substance use and crime outcomes [51]. In addition, for OMT patients where treatment focus may be primarily on the opiate use, simultaneously addressing stimulant use could be important [58].

Spending time with a substance using social network has previously been associated with increased likelihood of crime in substance users [3, 5]. We found that leaving a substance using network was associated with desistance, and that participants with no crime in the study period were also more likely to have had no substance using network in the study period. This is in line with findings that social identity and group membership in social networks that provide positive values and resources, may be important for both recovery and desistance from crime [10]. Further, the findings underscore the potential benefit of interventions that support development of new, non-using social networks [3, 59], for instance through social skills training [23]. Efforts to facilitate education, work and other activities with social contexts could provide both an arena for building social networks and meaningful daily activities which could have a positive effect on crime outcomes [51] and recovery from dependence [60].

We found an association between improved self-control score and desistance. There has been little research on the relationship between self-control and crime in substance using populations, although lower self-control has been associated with crime among substance using offenders [61, 62]. Our finding supports the potential utility of including components or interventions that could boost self-control in substance use treatment as well as in the criminal justice system. There is ongoing work in identifying and evaluating feasible interventions that could, in supplement to traditional treatment, directly or indirectly support self-control. For crime outcomes, self-control training in jail has been associated with both increases in self-control and desistance [63]. For substance use outcomes, self-control related interventions with promising results include goal management training, working memory training and cognitive bias modification [64, 65]. Finally, self-control must be seen in context with situational factors. Sufficiently motivating and realistic goals are a prerequisite for self-control, and as articulated by Burt: “Deliberation and PFC [prefrontal cortex] processing are luxuries reserved for those people who are not cognitively overloaded with, for example, survival efforts, threats, or emotional duress” (26, p64).

Among substance users both in and out of treatment, polysubstance use has been linked to crime [66]. In our study, the desisted crime group had a greater reduction in polysubstance use compared to the continued crime group, however, the results of the adjusted model were inconclusive. Our variable reflects the number of different substances used, but not the magnitude of the use (e.g. amounts, days of use per week). Thus we could not investigate whether reduction of substance use magnitude is effective in reducing crime, compared to complete abstinence [16], which is a question of great relevance when determining the goals of substance use treatment.

### Strengths and limitations

This study followed a relatively large cohort of participants who received comprehensive substance use treatment. The sample was naturalistic in the sense that the only exclusion criteria for participation were related to ethical considerations, such as the participants’ mental and physical well-being. Face-to-face interviews resulted in a low prevalence of missing data.

Attrition at T0 was mainly due to logistical challenges at the recruiting treatment sites (lack of resources among staff), still some of the most severe cases of substance use and dual diagnoses were not included in the study. Attrition at T1 was mainly due to the research group not reaching the participants (due to lack of updated contact information) or participants declining to participate. Agreeing to a follow-up interview was not an inclusion criterion at baseline. We cannot completely rule out that selection bias at T0 or T1 could have affected our results, we are however encouraged by two factors: First, sensitivity analyses detected no baseline differences between those included at follow-up and those lost to follow-up in demographics and the relevant variables for the analyses of this study. The only exception was that included participants reported a higher number of criminal acts at baseline, which is the opposite of what we would have expected if there was a serious selection bias favouring better-functioning participants at T1. Second, a methodologically relevant study of substance users (*n* = 654) found that results based on the 60% of the sample that were *easiest to reach* at follow-up were comparable to results based on 90–100% of the sample [36]. Taken together, this gives us the confidence to consider the study sample to be nationally representative of patients enrolled in inpatient treatment or OMT in Norway. We consider the results to be relevant for similar patient populations and settings.

Self-reported data on criminal activity have been found to be reliable [67–69]. Self-reported crime may have an advantage over use of official records that only reflect the instances where individuals were apprehended or convicted, and not the criminal acts they committed without these consequences [16, 67, 68].

The additional regression analysis comparing the no crime and continued crime groups should be interpreted with care. The total n in the comparison was low (*n* = 92) and the wide confidence intervals show that the estimates are uncertain.

For the additional measure of type and number of crimes, the assessed time-periods at baseline and follow-up differed (6 and 12 months). When reporting number of criminal acts, the difference in time-periods was addressed by calculating the average monthly acts before baseline and follow-up.

## Conclusions and implications

Participants starting inpatient and opioid maintenance treatment have great reductions in criminal activity 1 year after treatment initiation. Our data show that retention in treatment was important for reductions in crime, particularly among OMT patients, confirming the link between substance use treatment, recovery and desistance from crime. It becomes increasingly clear that, in addition to treatment retention and time in treatment, the content and quality of the treatment patients receive are crucial factors for explaining the individual differences in treatment outcome and recovery. A methodological challenge in observational research lies in finding reliable ways to measure both treatment factors and degree of recovery in a population where treatment interruptions, re-entries and change of treatment type are common [70].

Our findings show that social network and self-control are important outcomes of substance use treatment in relation to reducing crime. Treatment centres should, in addition to working towards reduced substance use, continue to strengthen the focus on improving the patients’ opportunity and ability to build a social network without substance use, and include specific cognitive programs or other training aiming at improvements in self-control. Stimulant-users in general are a high-risk group for continued criminal activity and may need strengthened targeted treatment interventions.

## Supplementary Information

**Additional File 1.** Supplementary tables and figures

## Data Availability

The datasets generated and/or analysed during the current study are not publicly available due to restrictions in the approval from the regional ethical committee, but are available from the corresponding author on reasonable request.

## Abbreviations

OMT: Opioid maintenance treatment
NorComt: Norwegian cohort of opioid maintenance treatment and other drug treatment
OR: Odds ratio
aOR: Adjusted odds ratio
CI: Confidence interval
EuropASI: European adaptation of the addiction severity index
BSCS: Brief self-control scale
PFC: Prefrontal cortex
