# Supplementary material for: Desistance from crime following substance use treatment: the role of treatment retention, social network and self-control
Source: BMC Psychiatry. 2021 Nov 12;21:563. doi: 10.1186/s12888-021-03518-2 (PMC8588672; doi:10.1186/s12888-021-03518-2)

Skjærvø, I., Skurtveit, S., Clausen, T. and Bukten, A. (2021)

Additional file 1

Supplementary Table 1 Baseline (T0) characteristics of participants included at follow-up (n=341) compared with participants lost to follow-up (n=207).

	Included n=341	Lost to follow-up n=207	p-value[a]
**Demographics**			
Women, n (%)	100 (29)	56 (27)	p = 0.57
Age, mean	34.0	33.3	p = 0.46
**Substance use pattern**			
**Substances/medications among the four most used 6 months before treatment, n (%)**			
Stimulants	229 (67)	142 (69)	p = 0.73
Alcohol	92 (27)	61 (29)	p = 0.53
Cannabis	222 (65)	148 (71)	p = 0.12
Illicit benzodiazepines	178 (52)	106 (51)	p = 0.82
Prescribed benzodiazepines	69 (20)	42 (20)	p = 0.99
Illicit opiates	170 (50)	106 (51)	p = 0.76
Prescribed opiates	74 (22)	41 (20)	p = 0.60
**Other substance use variables**			
Number of substances, mean (median)	4.3 (4.0)	4.6 (4.0)	p = 0.34[b]
Intravenous use 6 months, n (%)	219 (64)	129 (62)	p = 0.65
Previous treatment (≥ 3 months), n (%)	293 (88)	176 (86)	p = 0.56
**Crime variables**			
Any committed crime (yes/no), n (%)	217 (64)	131 (63)	p = 0.93
Number of criminal acts per month[c], mean (median)	19.7 (4.0)	12.1 (1.7)	**p = 0.024[b]**
**Other variables**			
Substance using social network, n (%)	180 (53)	97 (47)	p = 0.18
Ever served a prison sentence, n (%)	206 (60)	137 (66)	p = 0.18
Self-control (BSCS) score, mean	37.8	36.9	p = 0.27
Selected for in-patient treatment, n (%)	162 (48)	103 (50)	p = 0.61

Significant p-values in bold. [a] p-values from t-tests and chi-square tests. [b] Equal variances not assumed. [c] Among participants who had committed crime.

Supplementary Figure 1 Types of substances used at baseline (T0) for participants starting OMT and inpatient treatment (n=341).

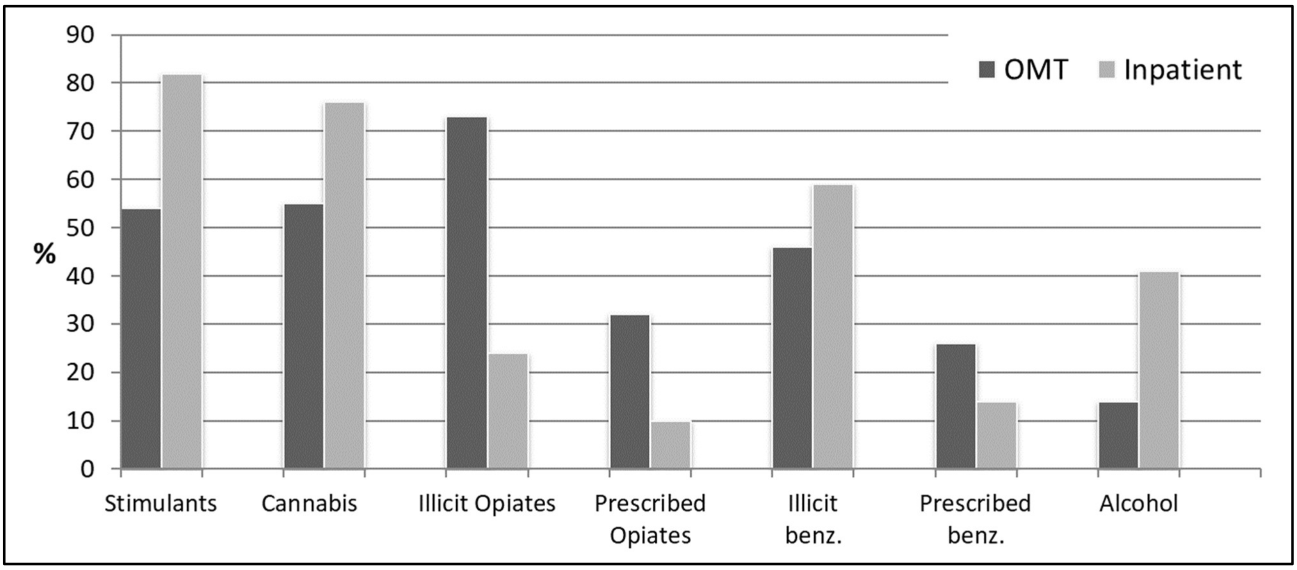

Benz. = Benzodiazepines.

Supplementary Figure 2. Percentages of participants (n=341) committing crime (yes/no) in the time-periods before T0 and T1.

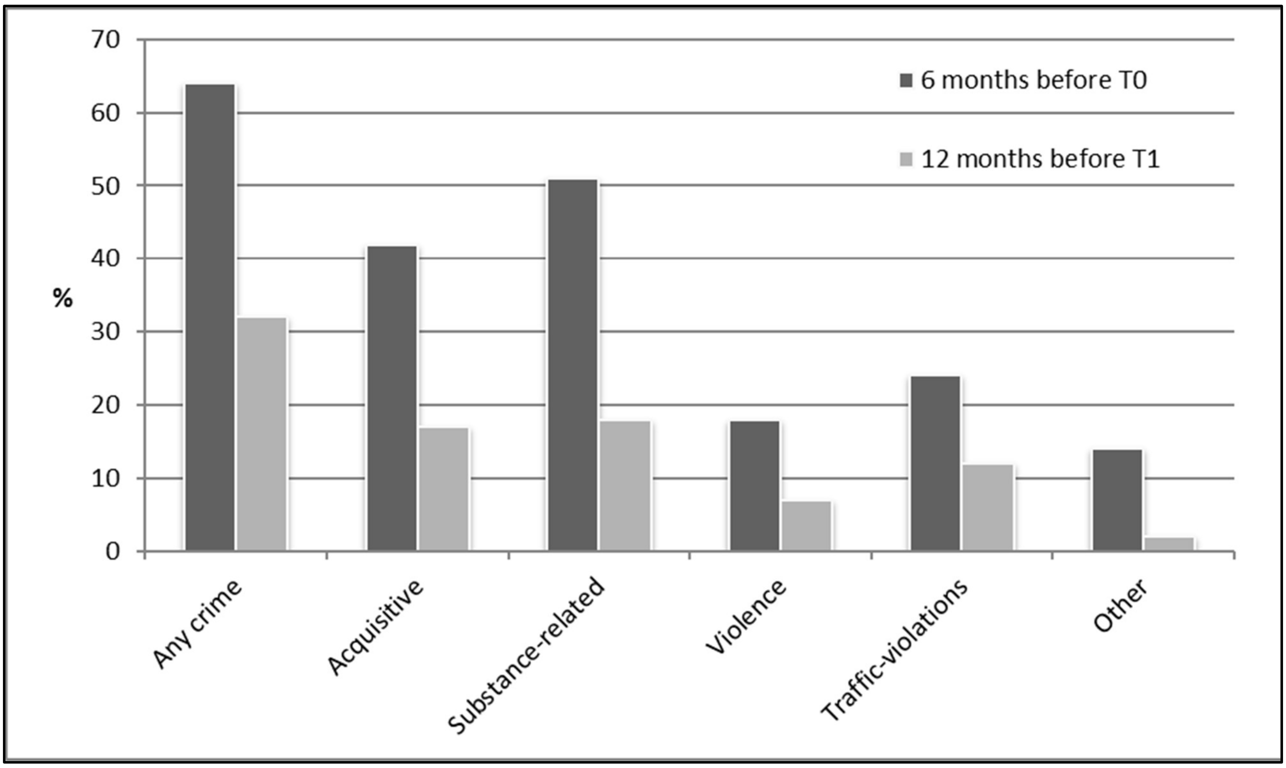

Supplementary Figure 3 Reductions in number of criminal acts from baseline (T0) to follow-up (T1), by treatment type and treatment status (n=63).

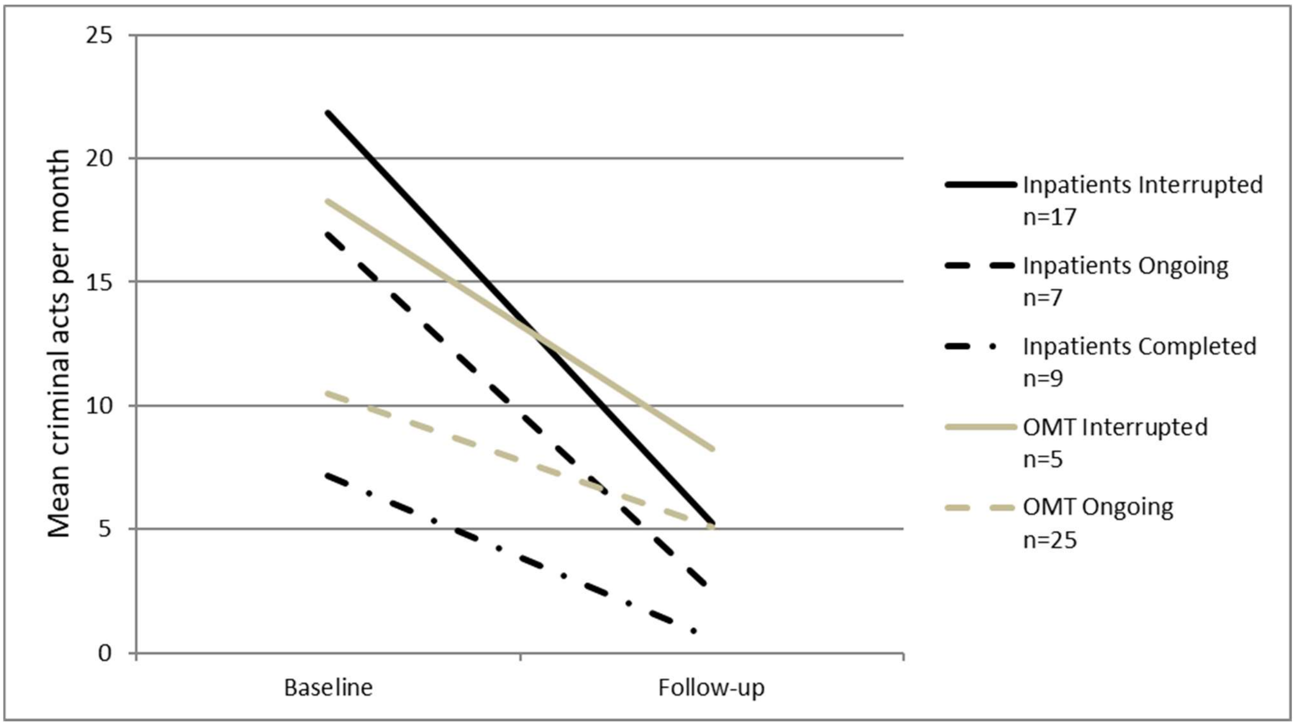

Supplement: Supplementary file 1 — Additional File 1. Supplementary tables and figures [file 12888_2021_3518_MOESM1_ESM.pdf]
